# Changing the South African national antiretroviral therapy guidelines: The role of cost modelling

**DOI:** 10.1371/journal.pone.0186557

**Published:** 2017-10-30

**Authors:** Gesine Meyer-Rath, Leigh F. Johnson, Yogan Pillay, Mark Blecher, Alana T. Brennan, Lawrence Long, Harry Moultrie, Ian Sanne, Matthew P. Fox, Sydney Rosen

**Affiliations:** 1 Department of Global Health, Boston University School of Public Health, Boston, MA, United States of America; 2 Health Economics and Epidemiology Research Office (HE2RO), Department of Medicine, Faculty of Health Sciences, University of the Witwatersrand, Johannesburg, South Africa; 3 Centre for Infectious Disease Epidemiology and Research, University of Cape Town, Cape Town, South Africa; 4 National Department of Health, Pretoria, South Africa; 5 National Treasury, Pretoria, South Africa; 6 Wits RHI, Faculty of Health Sciences, University of the Witwatersrand, Johannesburg, South Africa; 7 Clinical HIV Research Unit, Department of Medicine, Faculty of Health Sciences, University of the Witwatersrand, Johannesburg, South Africa; 8 Department of Epidemiology, Boston University School of Public Health, Boston, MA, United States of America; British Columbia Centre for Excellence in HIV/AIDS, CANADA

## Abstract

**Background:**

We were tasked by the South African Department of Health to assess the cost implications to the largest ART programme in the world of adopting sets of ART guidelines issued by the World Health Organization between 2010 and 2016.

**Methods:**

Using data from large South African ART clinics (n = 24,244 patients), projections of patients in need of ART, and cost data from bottom-up cost analyses, we constructed a population-level health-state transition model with 6-monthly transitions between health states depending on patients’ age, CD4 cell count/ percentage, and, for adult first-line ART, time on treatment.

**Findings:**

For each set of guidelines, the modelled increase in patient numbers as a result of prevalence and uptake was substantially more than the increase resulting from additional eligibility. Under each set of guidelines, the number of people on ART was projected to increase by 31–133% over the next seven years, and cost by 84–175%, while increased eligibility led to 1–26% more patients, and 1–17% higher cost. The projected increases in treatment cost due to the 2010 and the 2015 WHO guidelines could be offset in their entirety by the introduction of cost-saving measures such as opening the drug tenders for international competition and task-shifting. Under universal treatment, annual costs of the treatment programme will decrease for the first time from 2024 onwards.

**Conclusions:**

Annual budgetary requirements for ART will continue to increase in South Africa until universal treatment is taken to full scale. Model results were instrumental in changing South African ART guidelines, more than tripling the population on treatment between 2009 and 2017, and reducing the per-patient cost of treatment by 64%.

## Introduction

With over three million patients, the South African national public-sector antiretroviral treatment (ART) programme is the largest in the world [1]. It is also one of the few in Africa that is primarily funded from domestic resources, rather than international donor contributions [2,3]. The cost of this programme, which has risen steadily since its inception in 2004, has been and remains one of the major challenges confronting the South African government as it seeks to expand access to treatment and sustain or improve quality of service delivery. The National South African Department of Health (NDOH) faces the double challenge of expanding eligibility for and coverage of the HIV treatment programme while simultaneously reducing the per-person cost of treatment.

Since 2009, at the request of the NDOH, our research team at the University of the Witwatersrand in Johannesburg, South Africa and Boston University in the United States, together with collaborators from other institutions, has been working with the South African government to analyse the cost of the national HIV treatment programme and advise on the expected cost and impact of a series of new treatment guidelines issued by the World Health Organization (WHO) that aimed at providing better drugs while steadily raising the threshold of eligibility for ART. In order to estimate costs and impacts, we constructed a population-level, health-state transition model, the National ART Cost Model (NACM). The NACM captures both the guideline changes and the effects of implementing procurement, health systems, and technical improvements that help offset the additional cost of these guideline changes, such as introducing task-shifting to lower staff cadres and opening the antiretroviral drug market to international competition. Through regular updates to input prices and models of care, the NACM allows us to provide up-to-the-moment estimates to the National Department of Health as it considers new approaches and policies and secures the budgetary resources needed to sustain the programme.

In this article, we describe the NACM and how it has been used in the past seven years. When the NDOH initially requested assistance with budget estimates in 2009, the public-sector ART programme, which had been launched five years earlier, in April 2004, had initiated close to 1 million patients, of whom 919,923 were reported to still be in care in late 2009 [4]. Demand for treatment had increased rapidly to more than 300,000 new patients started on ART per year, placing tremendous pressure on funding and service delivery capacity. The generic antiretroviral drugs used in the programme were almost exclusively domestically produced, at prices that were higher than what other country programmes with access to international suppliers were paying. At the same time, government, clinicians, and civil society were debating a range of changes to the national ART guidelines, in response to new recommendations from WHO. The NACM provided the NDOH with both specific cost estimates and the ability to consider how variations in prices and approaches would affect the budget. Since then, the results of the model have contributed to revisions of national treatment guidelines in 2010, 2011, 2015, and 2016, and to nearly tripling the national budget allocation for ART over this period. This article reviews the methods and results of the NACM and discusses lessons learned from this collaboration.

Before describing the NACM itself, it is useful to be familiar with the history of South African treatment guidelines. Prompted by periodic revisions to the WHO’s guidance on antiretroviral treatment, between 2009 and 2016 the NDOH considered a range of changes to eligibility, drug regimens, and programmatic features. The NACM was used in each instance as a prospective tool for the projection of the total number of people on ART and total costs to be expected if the government was to implement each of these changes. **Table 1** summarises the timing and content of WHO recommendations, the corresponding NACM scenarios, and the ensuing changes to the South African ART guidelines; section 1 of the Appendix (S1 File) gives further details.

**Table 1:** Summary of WHO guideline recommendations, modelled scenarios and ensuing changes to the South African ART guidelines.

WHO guidelines		NACM scenario (date)	South African guidelines	
Date	Recommended changes^1^		Date	Mandated changes
		**SA 2004**	Apr 2004	See section 1 in the Appendix (S1 File)
**WHO 2010** Nov 2009 (rapid advice) [5]; July 2010 (full guidelines) [6]	*Adults*: Eligibility at 350 CD4 cells/μl; universal treatment^2^ for patients with active TB or hepatitis B (HBV) co-infection	**SA 2010** (Dec 2009)	Apr 2010 [7]	Adults: Eligibility at 350 CD4 cells/μl for patients with TB and pregnant patients; at 200 CD4 cells/μl for everyone else
	C*hildren*: Early paediatric treatment (< 2 yrs)			C*hildren*: Early paediatric treatment (< 1 yr)
	*Adults*: Replacement of d4T by TDF or AZT in first-line regimens			*Adults*: Replacement of d4T by TDF/ ABC in first-line regimens
	*Children*: Replacement of d4T by ABC in first-line regimens			*Children*: Replacement of d4T by ABC in first-line regimens
	Respective second-line regimens (TDF + 3TC/FTC + LPV/r or AZT + 3TC+ LPV/r)			Respective second-line regimens (TDF + 3TC/FTC + LPV/r or AZT + 3TC+ LPV/r)
				NIMART and ART at PHC level
	Fixed-dose combinations			Fixed-dose combinations and internationally competitive prices
		**SA 2011** (Dec 2009)	Aug 2011 [8]	Eligibility at 350 CD4 cells/μl for all patients
**WHO 2013** June 2013 [9]	*Adults*: Eligibility at 500 CD4 cells/μl; universal treatment for patients with TB or HBV co-infection, pregnant patients (PMTCT option B+), HIV-positive partners in serodiscordant couples, and pregnant and breastfeeding women	**SA 2015** (including 350, no B+; 350, B+; 500, no B+; 500, B+) (Mar 2014)	Jan 2015 [10]	*Adults*: Eligibility at 500 CD4 cells/μl; universal treatment for patients with HBV or TB and those who are pregnant, breastfeeding or within 1 year post-partum
	*Children*: Early paediatric treatment (< 5 yrs)			*Children*: Early paediatric treatment (< 6 yrs)
**WHO 2015** Sept 2015 [11]/ June 2016 [12]	Universal treatment for everyone	**SA 2016** (including 500, B+ (90-90-90); Universal treatment) (Nov 2015)	Sept 2016 [13]	Universal treatment for everyone
		(Nov 2015)	Jan 2016	Adherence guidelines (including adherence clubs)

*TB* tuberculosis; *HBV* hepatitis B; *d4T* stavudine; *TDF* tenofovir; *AZT* zidovudine; *3TC* lamivudine; *FTC* emtricitabine; *LPV/r* lopinavir boosted with ritonavir; *ABC* abacavir; *NIMART* nurse initiation and management of ART; *PHC* primary healthcare clinic; *yr* year.

^1^Throughout the table, eligibility also includes patients in WHO clinical stage 3 or 4 who have been eligible regardless of CD4 cell count since the WHO ART guidelines from 2003.

^2^Universal treatment signifies universal eligibility for treatment regardless of CD4 cell count for these patient groups.

## Methods

The NACM is a health-state transition model designed to estimate the number of patients on ART and the cost of treatment over eleven financial years (2010/11 to 2020/21). In this model, transitions between health states depend on patients’ age, CD4 cell count/ percentage, and time on treatment, and are based on data on treatment outcomes from large South African ART clinics (n = 24,244 patients), projections of patients in need of ART from existing models of the South African HIV epidemic, and cost data from bottom-up cost analyses in the same clinics that contributed outcome data.

### Model structure

The NACM uses inputs on a total population of HIV-infected individuals in need of ART as calculated by stand-alone HIV transmission models, the Actuarial Society of South Africa (ASSA) AIDS Model for the 2010 guidelines and the Thembisa model, for the 2013 and 2015 guidelines. Both models calculate the number of people living with HIV and the number of people initiating ART based on different assumptions of eligibility and uptake, with the resulting numbers of people in treatment being very similar between the transmission models and the NACM. Thembisa additionally calculates the number of people living with HIV who have been tested and know their status. Treatment uptake, or the relative rate of treatment initiation, was assumed to differ by CD4 cell count, as result of both demand- and supply-side factors (for more details please see Section 2.2 in the Appendix (S1 File)).

**Fig 1:**
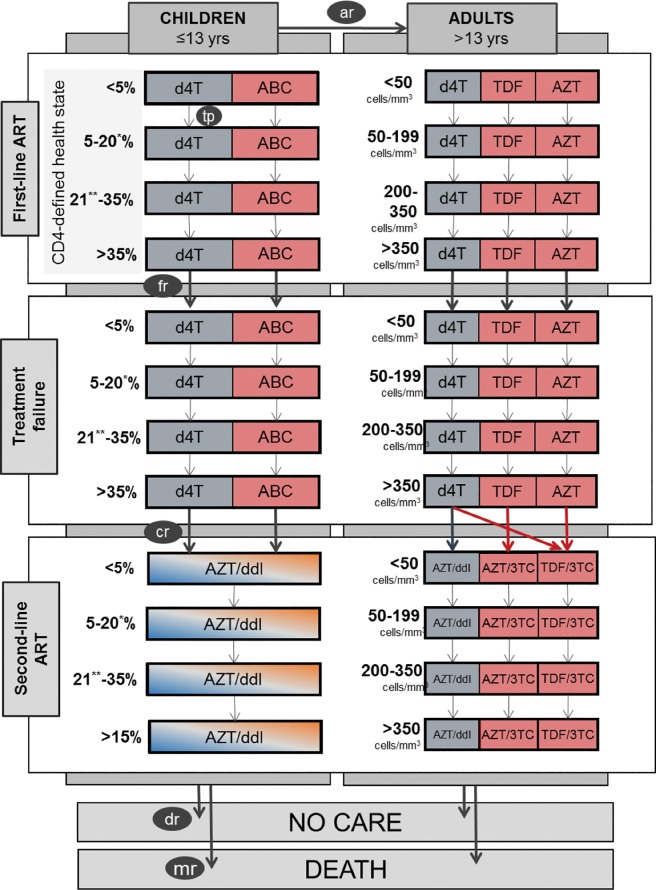
Health-state transition model. with ar: age rate, tp: transition probability, fr: failure rate, cr: rate of initiation of second-line ART, dr: default rate, mr: mortality rate. Blue arrows and boxes represent drug choices and transitions under the Old Guidelines scenario, red arrows and boxes represent the New Guidelines and Full WHO Guidelines scenarios. Drugs and transitions that are the same in all scenarios are represented in grey and white. For better legibility, rates are represented for the first row or column only; small black arrows represent movements in both directions; for drug choices, colours in the first row are representative of all rows; and only those drugs that change between scenarios are mentioned. *15% and **16% for children in the age group 6–13.

Based on a total population of HIV-infected individuals in need of ART as calculated by the transmission models, the NACM calculates the number of adults and children on first-line ART, the number who fail first-line ART, and the number who initiate second-line ART (Fig 1). For the analysis of the 2015 guidelines, a sub-population of patients failing second-line ART and initiating third-line ART was added. During each 6-month time interval, patients can either transition between these sub-populations, be lost from care, or die, according to estimated rates of ART coverage, treatment failure, loss to follow-up (LTFU), and death. Loss to follow up is calculated as net of patients returning to care at the same clinic after an interruption. The analysis of the 2010 guidelines did not capture the potential impact of increases in treatment coverage on HIV transmission, but the effect during our 7-year projection period was likely going to be negligible under any scenario that does not include universal treatment. (We however did include a transmission in the analysis of the 2013 and 2015 guidelines.)

Within each of the sub-populations, a health-state transition model calculates the number of patients in each CD4 cell count/ percentage stratum. For adults, CD4 strata are defined according to differences in mortality and LTFU and categorised as >350, 200–349, 50–199, and <50 cells/μl. For the analysis of the 2013 and 2015 guidelines, two additional strata were added (>500, 350–500 cells/μl). For children aged 6 to 13, CD4 strata are defined as >35, 15–35, 5–14, and <5%. CD4 strata for children <6 are CD4 >35, 20–35, 5–19, and <5%. The probabilities of transitioning between these strata vary by age, CD4 cell count, type of treatment, and, for adult ART, time on treatment. **Table 2** summarises the main NACM elements and their sources; more information on the model structure and the exact values of these elements can be found in section 2.4 of the Appendix (S1 File).

**Table 2:** Summary of model elements and data sources for NACM.

Model element	Data source
Number of adults initiating ART	ASSA2003 (WHO 2010 guidelines)/ Thembisa (WHO 2013 and 2015 guidelines)
Number of children initiating ART	Same as adults, plus additional assumptions regarding scale-up of Early Paediatric Treatment (EPT)
Mortality	TLC/ HSCC
Loss to follow-up	TLC/ HSCC
First-line treatment failure	TLC/ HSCC
Incidence of side effects necessitating drug change (adults only)	TLC data
Transition probabilities between health states	TLC/ HSCC
Outpatient cost	TLC/ HSCC

*ASSA2003* Actuarial Society of South Africa AIDS Model 2003; *TLC* Themba Lethu Clinic; *HSCC* Harriet Shezi Children’s Clinic

### Estimation of rates of death, loss to follow-up, and transition between CD4 cell count strata

We calculated rates of mortality, LTFU, treatment failure and development of side effects necessitating regimens changes amongst *adult* patients using data of 20,496 adult patients accessing ART at Themba Lethu Clinic (TLC), Helen Joseph Hospital, Johannesburg between April 2004 and May 2013- one of the largest adult ART clinics in South Africa. The clinic provides patients meeting government eligibility criteria with standard public sector ART. CD4 cell counts and HIV viral loads were monitored at roughly 6-monthly intervals after ART initiation. Data on mortality, loss to follow-up, and treatment failure rates amongst *paediatric* patients (14 years and younger) was based on an analysis of data from a longitudinal cohort of 3,748 paediatric patients accessing ART at Harriet Shezi Paediatric Clinic at Chris Hani Baragwanath Hospital, Gauteng, between April 2004 and May 2009. During this time period, the clinic initiated around 4,000 paediatric patients on ART, making the clinic one of the largest paediatric ART clinics in the world.

For the analysis of mortality, loss to follow-up (LTFU), and failure rates on first-line treatment, we included all adult patients on regimens not containing a protease inhibitor in the first-line sub-population, and all children on first-line regimens as specified in the relevant South African guidelines. Any previously virologically suppressed patient with two consecutive unsuppressed viral loads (>400 copies/ml for adults, >1000 copies/ml for children) was transitioned to the treatment failure (TF) sub-population, and any patient in the TF sub-population who switched to a protease-inhibitor containing regimen was transitioned to the second-line sub-population. Within each sub-population, we stratified all events (death, LTFU, treatment failure) by the patient’s last CD4 measure within a 6-month interval, and for adult patients on first-line ART, also by the patient’s time on ART. For patients missing a single 6-month CD4 cell count, we linearly interpolated this value from the adjacent CD4 cell counts. For children, we additionally corrected for a non-linear relationship between time and CD4 percentage during the first six months on treatment [14]. For the analysis of the 2015 guidelines, we additionally analysed rates of second-line ART treatment failure and coverage with third-line ART.

LTFU was defined as >6 months since last clinic visit and was net of patients returning back into care during the same time period in which they were lost. Mortality, LTFU, and transitions between health states were independent of whether d4T or TDF/ ABC were used. Deaths were considered only if the patient could be linked to an entry in the national death register. If patients were confirmed dead more than six months after their last visit to the clinic, they were classified as lost to follow up rather than dead, as their deaths would most likely have taken place after the patients had discontinued treatment. Data were collected by clinic staff for routine patient management purposes in electronic databases (TherapyEdge™ in Themba Lethu Clinic, Microsoft Access in Harriet Shezi Children’s Clinic) and abstracted, cleaned, and analysed using SAS versions 9.1 to 9.3.

Some of our estimates of mortality, loss and treatment failure within the sub-strata of CD4 count and time-on-treatment were unstable. In order to prevent undue influence of any single estimate on the results and to account for the variability in the estimates, we smoothed the estimated probabilities within CD4 count and time stratum. The details of this smoothing procedure as well as the resulting rates are given in Section 2.4 of the Appendix (S1 File).

### Cost data

Resource utilisation and unit cost data for adult ART patients were collected at the Themba Lethu Clinic (TLC) in Johannesburg between 2007 and 2009 and again in 2013 and were analysed from the provider perspective (Table 3) [15,16]. At each model run, data on outpatient resource use and treatment outcomes of 150 patients who had started ART at least 24 months before analysis were collected by reviewing patient records. Unit cost estimates were obtained from site-level financial records and updated to the latest relevant public-sector price lists for drugs, staff salaries and laboratory tests for each round of analysis. The cost of paediatric ART was assumed to be the same as for adult ART, with the exception of ARV drug costs, for which we used the mean weight of children in care at Harriet Shezi Children’s Clinic (HSCC) as a guide to average drug dosages by age group (<12 months, 1–5 years, 6–13 years).

**Table 3:** Cost inputs.

SA 2010 and 2011 [2009 USD]						
***A*. *SA 2004***						
**Annual per patient cost (Adults)**						
First-line treatment	672					
First-line treatment failure	662					
Second-line treatment	1,531					
**Annual per patient cost (Children)**			**Initiated at ≤3 yrs**		**Initiated at >3 yrs**	
	**<12 mts**	**12–35 mts**	**3–5 yrs**	**6–13 yrs**	**3–5 yrs**	**6–13 yrs**
First-line treatment	539	675	796	808	631	686
First-line treatment failure	575	712	835	847	695	723
Second-line treatment	815	994	799	903	1,137	1,190
***B*. *SA 2010 and SA 2011***						
**Annual per patient cost (Adults)**	**TDF-containing regimens**	**AZT-containing regimens**				
First-line treatment	799	703				
First-line treatment failure	802	694				
Second-line treatment	1,235	1,139				
**Annual per patient cost (Children)**			**Initiated at ≤3 yrs**		**Initiated at >3 yrs**	
	**<12 mts**	**12–35 mts**	**3–5 yrs**	**6–13 yrs**	**3–5 yrs**	**6–13 yrs**
First-line treatment	631	872	1,020	1,157	854	1,034
First-line treatment failure	667	967	1,061	1,200	946	1,076
Second-line treatment	815	994	799	903	1,137	1,190
**SA 2015 [2014 USD]**	** **					
**Annual per patient cost (Adults)**						
First-line treatment	376					
First-line treatment failure	336					
Second-line the treatment therapy	717					
**Annual per patient cost (Children)**	**<12 mts**	**12–35 mts**	**3–5 yrs**		**6–13 yrs**	
First-line treatment	370	342	114		402	
First-line treatment failure	227	112	145		458	
Second-line treatment	545	544	453		533	
**SA 2016 [2015 USD]**			** **	** **	** **	** **
**Annual per patient cost (Adults)**	**Standard model of care**	**With adherence clubs**				
First-line treatment	248	243				
First-line treatment failure	248	248				
Second-line treatment	495	495				
Second-line treatment failure	754	754				
Third-line treatment	1,372	1,372				
**Annual per patient cost (Children)**			**Initiated at ≤3 yrs**		**Initiated at >3 yrs**	
	**<12 mts**	**12–35 mts**	**3–5 yrs**	**6–13 yrs**	**3–5 yrs**	**6–13 yrs**
First-line treatment	263	237	240	264	220	256
First-line treatment failure	293	258	263	294	236	283
Second-line treatment	475	503	478	497	510	541

*USD* US dollars; *mts* months; *yrs* years; *TDF* tenofovir; *AZT* zidovudine

Average cost per patient was varied in the model between types of treatment (first-line treatment, first-line treatment failure, second-line treatment), drug regimens, and age groups (defined as adults vs. children, and for children depending on current age and age at treatment initiation, as shown in **Table 1**), but was assumed not to vary by CD4 cell count. For the analysis of the 2010 WHO guidelines, we used Clinton Foundation ceiling prices from August 2009 for ARV costs [17], and replaced all physicians by senior-level nurses and all pharmacists by pharmacy assistants, while allowing for supervision time by the replaced cadre, for the reduced cost scenario. For the analysis of the 2015 guidelines, we assumed a 20% reduction in staff costs under adherence clubs, or a 6% reduction in annual costs overall, based on an economic analysis of the adherence clubs in Khayelitsha, South Africa [18].

We calculated total cost by multiplying the numbers of patients in each sub-population in each 6-month model cycle by the appropriate average 6-month unit cost. Deaths and losses were assumed to occur on average in the middle of the cycle and thus only incur 50% of the half-year unit cost in the cycle in which they occurred. All costs are presented undiscounted and adjusted to the year that the analysis was done, in order to preserve consistency with the relevant cost inputs at the time of the decision, using the relevant average ZAR: USD exchange rate for the year in question.

### Analytical steps

For each analysis informing changes to the South African guidelines we followed the same process: Firstly, we used programme data to estimate or update our estimates of the number of people currently on treatment as produced by the transmission models. We then estimated the impact that each guideline change would have on the number of patients initiating and on ART. Based on this, we estimated the cost of treating the total cohort of patients on ART, and the incremental cost of treating the additional patients added by a change in treatment criteria, as well as any changes to drug regimens. Finally, we calculated the impact on total and incremental costs of a number of cost-saving policies, such as the use of lower levels of staff cadres or the opening of the drug market to international competition. The presentation of results in the next section follows this process for each of the guideline changes we considered.

Use of data was approved by the Institutional Review Board of Boston University School of Public Health (protocol number H-28413) and the Human Research Ethics Committee of the University of the Witwatersrand (protocol number MO60626).

## Results

The primary outputs of the NACM are the numbers of patients expected to initiate ART and remain on treatment each year (Table 4) and the cost of the treatment programme (Table 5). Results are reported below for each major guideline change, as described above in Table 1.

**Table 4:** Total number of patients on ART [thousands].

Scenario	Total number of patients to be initiated on ART	Total patients projected to remain on ART (% change on previous guidelines)		
	(% change on previous guidelines)			
	Years	Start year	End year	% change over time
**2010 WHO guidelines**	**2010–2016**	**2010**	**2016**	** **
SA 2004	2,928	1,333	3,061	130%
SA 2010	3,334 (14%)	1,409 (6%)	3,491 (14%)	148%
SA 2011	3,596 (23%)	1,655 (24%)	3,855 (26%)	133%
**2013 WHO guidelines**	**2014–2020**	**2014**	**2020**	** **
350, no B+	1,929	3,119	5,087	63%
350, with B+	1,979 (3%)	3,132 (0.4%)	5,127 (1%)	64%
500, no B+	2,171 (13%)	3,303 (6%)	5,260 (3%)	59%
500, with B+	2,200 (14%)	3,315 (6%)	5,282 (4%)	59%
**2015 WHO guidelines**	**2016–2020**	**2016**	**2020**	** **
500, with B+ (90-90-90)	3,936	4,174	5,448	31%
Universal Treatment	4,740 (20%)	4,301 (3%)	5,864 (8%)	36%
** **	**2016–2034**	**2016**	**2034**	** **
500, with B+ (90-90-90)	9,504	4,174	6,165	32%
Universal Treatment	7,741 (-19%)	4,301 (3%)	5,468 (-11%)	27%

**Table 5:** Total outpatient cost by scenario [million USD].

Scenario	Full unit cost (% change on previous guidelines)				Reduced unit cost (% change on previous guidelines)				
	Start year	End year	Change over time	Total	Start year	End year	Change over time	Total	Change on Full cost
** **	** **	** **	** **	** **	** **	** **	** **	** **	** **
**2010 WHO guidelines [2009 USD]**	**2010**	**2016**	** **	** **	**2010**	**2016**	** **	** **	** **
***Definition of unit cost***	***Standard model of care***				***With task-shifting and fixed-dose combinations***				*** ***
SA 2004	1,002	2,477	147%	12,293	633	1,570	148%	7,775	-37%
SA 2010	1,084 (8%)	2,982 (20%)	175%	14,358 (17%)	676 (7%)	1,939 (24%)	187%	9,190 (18%)	-36%
SA 2011	1,267 (26%)	3,287 (33%)	159%	16,281 (32%)	786 (24%)	2,129 (36%)	171%	10,370 (33%)	-36%
**2013 WHO guidelines [2014 USD]**	**2014**	**2020**	** **	** **	** **	** **	** **	** **	** **
350, no B+	1,200	2,270	89%	11,666					
350, with B+	1,205 (0.4%)	2,285 (0.7%)	90%	11,748 (0.7%)					
500, no B+	1,271 (5.9%)	2,341 (3.1%)	84%	12,231 (4.8%)					
500, with B+	1,275 (6.3%)	2,350 (3.5%)	84%	12,285 (5.3%)					
**2015 WHO guidelines [2015 USD]**	**2016**	**2020**	** **	** **	**2016**	**2020**	** **	** **	** **
***Definition of unit cost***	***Standard model of care***				***With adherence clubs and home-based ART***				*** ***
500, with B+	1,031	1,423	38%	6,154	1,013	1,390	37%	6,020	-2%
Universal Treatment	1,063 (3%)	1,529 (7%)	44%	6,544 (6%)	1,044 (3%)	1,493 (7%)	43%	6,400 (6%)	-2%
** **	**2016**	**2034**	** **	** **	**2016**	**2034**	** **	** **	** **
500, with B+	1,031	2,050	99%	30,714	1,013	2,015	99%	30,081	-2%
Universal Treatment	1,063 (3%)	1,818 (-11%)	71%	30,409 (-1%)	1,044 (3%)	1,786 (-11%)	71%	29,778 (-1%)	-1%

### WHO 2010 and SA 2010 and 2011 Guidelines

If the guidelines prevailing in 2009 (SA 2004) guidelines had remained in place, we projected that, 2.9 million new patients would have initiated ART between financial years 2010/11 and 2016/17, and the number of people on ART would have increased from 1.333 million patients in 2010 to 3.061 million patients in 2016, a 2.3-fold increase (**Table 4**). Under two variations on the 2010 WHO guidelines, SA 2010 and SA 2011, the total numbers of patients expected to initiate ART in this period were 3.3 million and 3.6 million, respectively, a 1.14- and 1.23-fold increase. We thus estimated that patient numbers would more than triple over seven years in all three scenarios, and that the differences between the scenarios- an increment of about 400,000 patients between each one- were much smaller than the difference between the baseline number on treatment and the number projected even without guideline changes. In other words, the increase in numbers on ART over time due to increasing HIV prevalence and improved programme coverage dwarfed the increase due to expanded eligibility represented by each scenario.

In the analysis of the impact of the 2010 WHO guidelines, the annual cost of the programme in 2010/11 was estimated to be between USD 1.1 and 1.5 billion, if staff and drug cost were unchanged (**Table 5**). This cost was predicted to increase by between 149% and 180% over seven years under each scenario as a result of increasing numbers of patients moving to second-line ART over time and, in some scenarios, to more expensive first-line regimens. As with the numbers of people on ART, most of this increase (164% to 180%) was due to the increasing numbers in need of ART, rather than to expanded eligibility or improved first-line drugs (20% to 36%). We projected that if antiretroviral drugs were accessed at current Clinton Foundation ceiling prices, and as fixed-dose combinations wherever possible, however, the total projected outpatient cost of the programme would decrease by between 22% and 23% depending on scenario, and if this was combined with task-shifting, total cost would decrease by between 36% and 37%, while reaching between 14% and 26% more patients.

### WHO 2013 and SA 2015 Guidelines

In the NACM analysis of how the 2013 WHO guidelines would affect patient numbers and costs in South Africa, the total number of patients remaining on ART increased by between 59% and 63% over the 7-year projection period, but by only 1% to 6% between different eligibility scenarios. We were surprised to find that by the time of the analysis in 2013, the projected number of people expected to initiate ART between 2014/15 and 2020/21 was lower in each scenario than the number previously projected in 2010, due to the effect of three years of implementation of eligibility at 350 cells/μl in the 2011 South African guidelines, which dramatically reduced the number of people with CD4 cell counts of <350 cells/μl not yet on treatment. This progress toward high coverage levels in the South African ART programme was expected to diminish the numbers of patients still needing to initiate treatment per year starting as early as 2015. Of note is that the decision to make all pregnant women eligible for life-long ART regardless of CD4 cell count (PMTCT option B+) increased the projected number of patients initiating ART only very marginally (by 3% and 1% at eligibility of 250 and 500 CD4 cells/ microl for all other adults, respectively).

The analysis also showed that while total cost would almost double over the 7-year projection period under any scenario of eligibility, it would increase only marginally through the elevation of the eligibility threshold from 350 to 500 cells/μl (an increase of 4.8%), and even less so through the additional implementation of PMTCT Option B+ (an additional increase of between 0.5 and 0.7%).

### WHO 2015 and SA 2016 Guidelines

The analysis of the 2015 WHO guidelines, in contrast to previous analyses, showed that implementation of universal test-and-treat to reach the UNAIDS 90-90-90 targets would increase the number of patients expected to initiate ART over the short term. Over the longer term, both the numbers of people initiating and remaining on ART per annum declined in comparison to eligibility at 500 cells/μl, by 19% and 11%, respectively.

The analysis also showed that, as with the number of people on ART, costs would increase slightly through the implementation of universal treatment. The increase over time was markedly less than in the previous analyses, due to the high coverage level achieved by the South African ART programme that were mentioned above, and could be more than reversed through the implementation of adherence clubs, in essence a form of task-shifting from nurses to peer counsellors who are tasked with reviewing and dispensing ARVs to stable adult patients on first-line regimens, who comprise the majority of patients in the treatment programme. By 2024, numbers on ART as well as total cost would begin to decline largely as a result of the impact of a high level of ART coverage on new infections (treatment as prevention) and, to a much lesser extent, the scale-up of other prevention interventions. Overall, total costs over 20 years would be 11% less under universal treatment than under eligibility at 500 cells/μl.

### Resulting changes to the HIV Conditional Grant budget

At the outset of our engagement with the South African government in early 2009 the process of setting a budget for the ART programme at the national level was somewhat haphazard, with targets and unit costs set by provinces based on past budgets or assumptions. While strategy such as the National Strategic Plan for HIV and AIDS & STIs 2007–2011 had been based on detailed budget analyses using population data based on epidemiological models, unit costs based on cost analyses, and coverage targets agreed on by policy makers, the annual Conditional Grant budgets that were submitted by the NDOH to Treasury were principally based on provincial plans that contained almost no analysis. The National ART Cost Model added to this the possibility of analysing both national- and provincial-level cost based on detailed data regarding the target population, population remaining in care, and unit costs.

**Fig 2:**
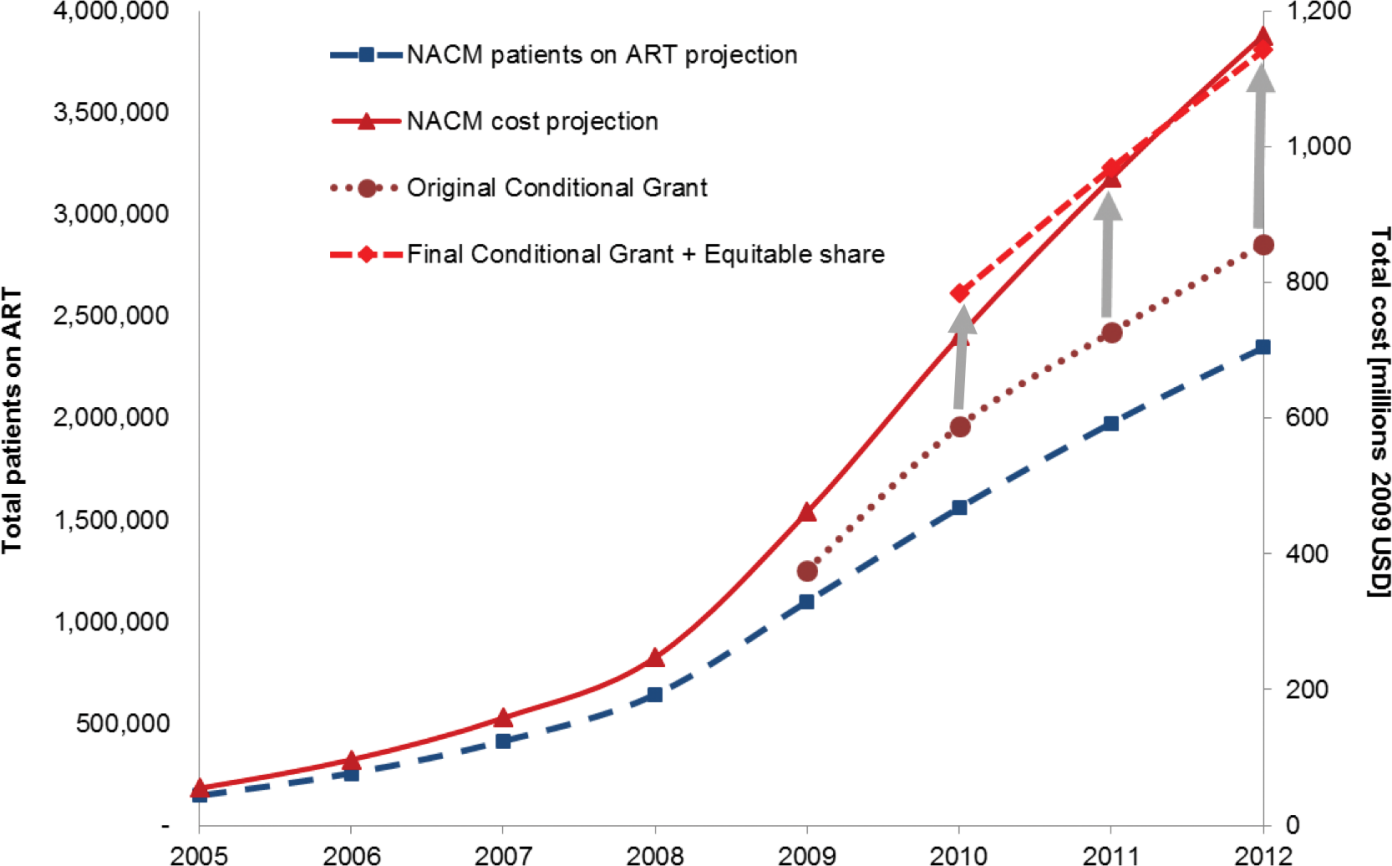
Development of HIV Conditional Grant amounts (2009/10 to 2011/12), in comparison to total patient numbers and total ART cost as calculated by NACM.

The incorporation of the National ART Cost Model into the budget planning process gave policy makers in the National Department of Health the ability to make the case for additional funding required to increase eligibility and offer better drugs, while it supplied decision-makers in the National Treasury with the confidence to commit to increasing the HIV Conditional Grant. Based on the first analysis, of the 2010 WHO guidelines, this grant was increased by 30% over the originally planned amount in 2010/11, and by 100% in 2011/12. Fig 2 summarises the relationship between the originally planned CG amounts, the numbers of patients on ART and total cost of the ART programme projected by the NACM, and the final resulting CG amounts for the financial year 2009/10 and 2011/12.

**Fig 3:**
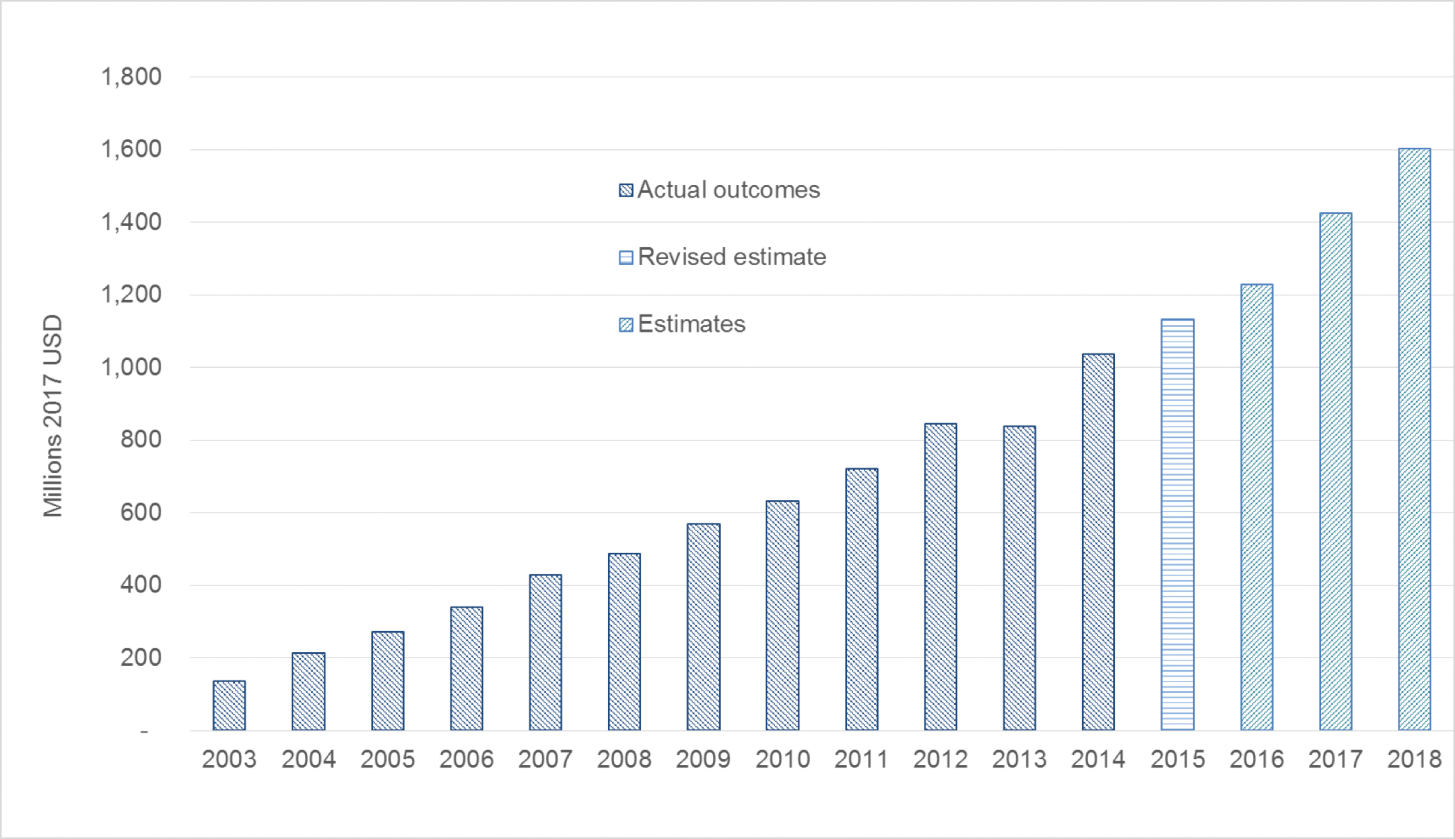
Government expenditure on HIV (2003/04 to 2018/19) [2017 ZAR]. Data updated based on [18], sources: Estimates of Provincial Expenditure/ Estimates of National Expenditure 2004/5 to 2014/15, Medium Term Policy Statements, Division of Revenue Bill/ Acts; all from National Treasury.

The HIV Conditional Grant budget kept increasing between the 2012/13 and 2017/18 financial years, again based in part on the results of the NACM, bringing the total increase in the government’s spending on HIV in real terms to 951% over the life of the ART programme (since 2003/4), or 193% since 2008/09, the last year before the NACM was used to define the ART budget (Fig 3) [19].

## Discussion

The National ART Cost Model allowed us to estimate, prior to policy adoption, the likely impact and cost to the largest ART programme in the world of incorporating into national guidelines the changes recommended by the World Health Organization between 2009 and 2015. For each set of guidelines, the increase in cost due to increased eligibility for ART was dwarfed by the increase in total cost resulting from the growth in the population in need of ART, a trend which started to level off around 2014 due to the high coverage levels of the treatment programme overall. In analysing the 2010 and the 2015 WHO guidelines, our model indicated that the projected increases in treatment cost could be offset in their entirety by the introduction of cost-saving measures, such as opening the drug tenders for international competition bound to Clinton Foundation ceiling prices and introducing task-shifting from doctors to nurses and ultimately, in the form of adherence clubs, from nurses to peer counsellors. Under universal treatment we were able to, for the first time, predict a reduction of the annual cost of the treatment programme from about 2024 onwards.

Our analysis incorporates a number of innovations, the most important being the addition of time on treatment to the analysis of transition probabilities between health states for adults on first-line treatment who constitute the majority of the model population. Our analysis of survival in care and CD4 cell count development for adults showed that transitions between CD4 cell count strata, mortality, and loss to follow up under first-line ART depend on time on ART as well as on the patient’s current CD4 cell count. Finally, in contrast to a once-off published cost analysis, the structure of the NACM allows regular updates to input prices and models of care. The resulting average cost of ART per year and patient type is thus relevant to the current moment in time and reflects the maturation of the treatment cohort by representing the currently relevant distribution into adults and children, new and established patients, and specific first and second line regimens.

There are a number of limitations to the study as well. Our model was set up to make maximum use of patient-level laboratory and outcome data, allowing us to extrapolate mortality, LTFU, and treatment failure at a high level of detail, while cost was differentiated by type of treatment and age group only and assumed to be uniform across CD4 cell counts and time on treatment. A limited number of studies suggest that per patient costs tend to be lower when ART is initiated at higher CD4 cell counts [20,21], which means that our results especially for the SA 2010 and SA 2011 scenarios might have been an overestimation. We also included outpatient costs only; some of these costs could have been offset by savings in inpatient costs as patients initiated at higher CD4 cell counts and avoided the higher inpatient care costs associated with lower CD4 cell count strata. Our own work has shown that the inpatient cost of patients on ART tends to be higher than that of patients in pre-ART care, so this cost saving would likely be limited, and unlikely to offset the increased cost due to higher eligibility [22]. On the other hand, our assumptions of ART initiation rates at CD4 cell count levels for which no routine data was available could have been too cautious. We also sourced our cost estimates from only two clinics, both of which are large and possibly not representative of resource use at more standard sites. This could have led to an overestimation of costs, since these sites might be better equipped and staffed than standard sites, or to an underestimation, since large clinics might experience economies of scale. Lastly, for the 2010 guidelines analysis we did not include the potential impact of treatment on HIV transmission; this impact was however included in the other two sets of guideline analyses.

While the NACM and its results strengthened the hand of the policy makers in the South African government, it is important to note that the NACM was only one of the factors in this process. A major contributor to this change in policy was the strong leadership exerted by the current Minister of Health and senior managers within the NDOH. Work by the Clinton Health Access Initiative in 2010 was instrumental in making the case for the pegging of tender prices to the lowest internationally available prices, as well as assembling the resulting price reference list. Staff within the Affordable Medicines Directorate within the NDOH led the antiretroviral tender negotiations that resulted in a decrease of a mean of 50% in the price of single ARVs in the 2010 tender and further reductions in recent tenders, resulting in a 63% and 68% reduction of the annual per patient cost of adult first-line and second-line treatment, respectively (see Table 3), and allowing South Africa to access ARVs at the lowest prices internationally. By providing cost and impact estimates that were accepted by all stakeholders, however, it was ultimately the NACM that convinced policy makers that South Africa both should and could adopt the changes proposed.

## Supporting information

S1 File Appendix. (PDF)
