# Supplementary material for: Changing the South African national antiretroviral therapy guidelines: The role of cost modelling
Source: PLoS One. 2017 Oct 30;12(10):e0186557. doi: 10.1371/journal.pone.0186557 (PMC5662079; doi:10.1371/journal.pone.0186557)
Supplement: S1 File — (PDF) [file pone.0186557.s001.pdf]

S1 File

Appendix for "Changing the South African national antiretroviral therapy guidelines: The role of cost modelling"

Gesine Meyer-Rath[1,2], Leigh F. Johnson[3], Yogan Pillay[4], Mark Blecher[5], Alana T Brennan[1,2], Lawrence Long[2], Harry Moultrie[6], Ian Sanne[2,7], Matthew P. Fox[1,2,8], Sydney Rosen[1,2]

[1] Department of Global Health, Boston University School of Public Health, Boston, US
[2] Health Economics and Epidemiology Research Office (HE[2]RO), Department of Medicine, Faculty of Health Sciences, University of the Witwatersrand, Johannesburg, South Africa
[3] Centre for Infectious Disease Epidemiology and Research, University of Cape Town, Cape Town, South Africa
[4] National Department of Health, Pretoria, South Africa
[5] National Treasury, Pretoria, South Africa
[6] Wits RHI, Faculty of Health Sciences, University of the Witwatersrand, Johannesburg, South Africa
[7] Clinical HIV Research Unit, Department of Medicine, Faculty of Health Sciences, University of the Witwatersrand, Johannesburg, South Africa
[8] Department of Epidemiology, Boston University School of Public Health, Boston, US

1. Details of modelled guideline changes

2010 WHO guidelines

Eligibility at 350 CD4 cells/µl for adults; early paediatric treatment (all children <2 yrs of age); better first-line drugs to replace d4T

The WHO ART guidelines from 2010 (issued as Rapid Advice in Sept 2009, and as formal guidelines in June 2010) increased the immunological threshold for eligibility from <200 CD4 cells/µl to <350 CD4 cells/µl and replaced stavudine (d4T) with tenofovir (TDF) in first-line ART for newly initiated adults [3]. Proposed changes to the paediatric ART guidelines included initiating on treatment all children <12 months who test positive by HIV PCR, regardless of clinical or immunological status (early paediatric treatment), and the replacement of stavudine with abacavir (ABC) in first-line regimens for newly initiated children.

For the analysis leading to the 2010 South African guidelines, we analysed numbers of people initiating ART, their survival in care and the resulting cost under three scenarios (Table 1). The ***Old South African (SA) Guidelines*** scenario kept the same eligibility thresholds and ART regimens for adults and children as the 2004 South African ART guidelines [3]. Under the ***New SA Guidelines*** scenario, the adult eligibility threshold was raised to <350 cells/µl for patients with TB and for pregnant women while continuing to initiate all other adults at <200 cells/µl, and treatment was initiated for all

HIV-positive babies <12 months of age immediately after the first positive PCR (Early Paediatric Treatment). The New SA Guidelines scenario also replaced d4T in first-line regimens with TDF for adults and abacavir (ABC) for infants for newly initiated patients or those experiencing severe d4T toxicity. Finally, the ***Full WHO Guidelines*** scenario increased the eligibility threshold to <350 cells/µl for all adults, while keeping paediatric eligibility and all regimens the same as in the New SA Guidelines scenario. We assumed that survival in care would change between scenarios as a function of the higher eligibility threshold (calculated based on CD4 cell count dependent transition probabilities), but would be the same between different drug regimens; both eligibility and drug regimen changes however impacted on cost.

Within each of the scenarios, we also examined two other proposed changes: the impact of task-shifting, defined as ART initiation and management by nurses under physician supervision and the dispensation of ART by pharmacy assistants under pharmacist supervision; and the impact of replacing the then existing system of antiretroviral drug procurement via government tenders that favour domestic production with drugs sourced internationally at ceiling prices negotiated by the Clinton Foundation.

S1 Table: Scenarios of analysis for 2010 guidelines

**Scenario**	**SA 2004**	**SA 2010**	**SA 2011**
Eligibility criterion			
**Adults**	<200 CD4 cells/µl<br>or WHO stage 4	For HIV/ TB co-infected and pregnant patients:<br><350 CD4 cells/µl<br>For all other patients:<br><200 CD4 cells/µl<br>or WHO stage 4	<350 CD4 cells/µl<br>or WHO stage 4
**Children**	15% to 20% of total lymphocyte count or WHO stage 3/4	After first positive PCR in 1st year of life, regardless of CD4 cell percentage or WHO stage (Early Paediatric Treatment)	
Drug regimens			
**Adults**			
First-line	d4T + 3TC + EFV or NVP	For all new initiates and those with d4T toxicity:<br>TDF + 3TC + EFV or NVP<br>For all else:<br>d4T + 3TC + EFV or NVP	
Second-line	AZT + ddI+ LPV/r	For those failing d4T- or AZT-containing regimens:<br>TDF + 3TC + LPV/r<br>For those failing TDF-containing regimens:<br>AZT + 3TC + LPV/r	
**Children**			
First-line	*<3 yrs:* d4T + 3TC + LPV/r<br>*>3 yrs:* d4T + 3TC + EFV or NVP	*<3 yrs:* ABC + 3TC + LPV/r<br>*>3 yrs:* ABC + 3TC + EFV or NVP	
Second-line	*<3 yrs:* AZT + ddI + NVP<br>*>3 yrs:* AZT + ddI + LPV/r		

2013 WHO guidelines

Eligibility at 500 CD4 cells/µl and universal treatment for patients with TB and those who are pregnant*; *early paediatric treatment (all children <5 yrs)

In 2012, as a precursor to the WHO guidelines issued in June 2013, discussions within government and the South African HIV community at large began about shifting the general ART eligibility threshold to 500 cells/µl for adults, and increasing the age of eligibility for early paediatric treatment from 1 to 5. We modelled the impact of this, together with the impact of replacing the then current 5-pills-a-day regime by a 1-pill-a-day fixed-dose combination formulation of the same drugs for first-line adult treatment. On request of the DOH, we modelled the following four options:

- Eligibility at 350 CD4 cells/µl, no PMTCT option B+ (initiation of universal treatment for life of pregnant women)
- Eligibility at 350 CD4 cells/µl, with B+
- Eligibility at 500 CD4 cells/µl, no B+
- Eligibility at 500 CD4 cells/µl, with B+.

2015 WHO guidelines

Universal treatment for everyone

In September 2015, WHO recommended the elimination of eligibility thresholds altogether, and the initiation of ART in everyone testing HIV positive regardless of CD4 cell count (termed 'universal treatment'). Since 2013, we have modelled the potential budget implications of universal treatment under various assumptions of uptake. In the analysis presented here, we extended the analysis to a 20-year framework as part of our work for the South African HIV Investment Case, and added the impact of providing care for stable adult patients on first-line therapy in adherence clubs led by peer counsellors rather than 1-on-1 nurse encounters.

2. Data sources and assumptions

2.1 Population in need of ART

The model calculates the cost of treating all patients in need of ART between 2010/11 and 2020/21, including those patients already in care by the beginning of the financial year 2010/11, in April 2010 (965,005 adults and 336,267 children). Data on the population in need of and initiating ART were obtained from a number of HIV transmission models that were parameterised for South Africa and regularly updated as new programme data became available.

The models used in each analysis were as follows:

- **2010 WHO guidelines**: The population in need of and initiating ART was based on an adaptation of the Actuarial Society of South Africa (ASSA) AIDS model from 2005 (ASSA2003), developed by the Centre for Actuarial Research, University of Cape Town [4,5], together with an add-on model that calculateda breakdown of the population assumed to initiate ART by CD4 cell count, allowing patients to enter care in a specified CD4 count stratum [6]. This model allows for a

change in the number of patients initiating care if immunological eligibility is defined at <350 CD4 cells/µl rather than <200 cells/µl.

- **2013 WHO guidelines:** The population in need of ART came from Thembisa, a synthesis of the ASSA AIDS Model and a number of other models developed by the Centre for Infectious Disease Epidemiology and Research, University of Cape Town [7,8].
- **2015 WHO guidelines**: This analysis was part of the South African HIV Investment Case [9,10,11]. As all other target populations for the Investment Case, the population in need of and initiating ART was based on an updated version of the Thembisa model.

Figure 1 illustrates the fit of the two models to data from existing South African surveys, in this case of antenatal HIV prevalence. ASSA2003 was calibrated using data published up to 2003, while Thembisa v2.4 was calibrated using data published up to 2012.

S1 Figure: Calibration of ASSA2003 and Thembisa to existing South African surveys of antenatal HIV prevalence

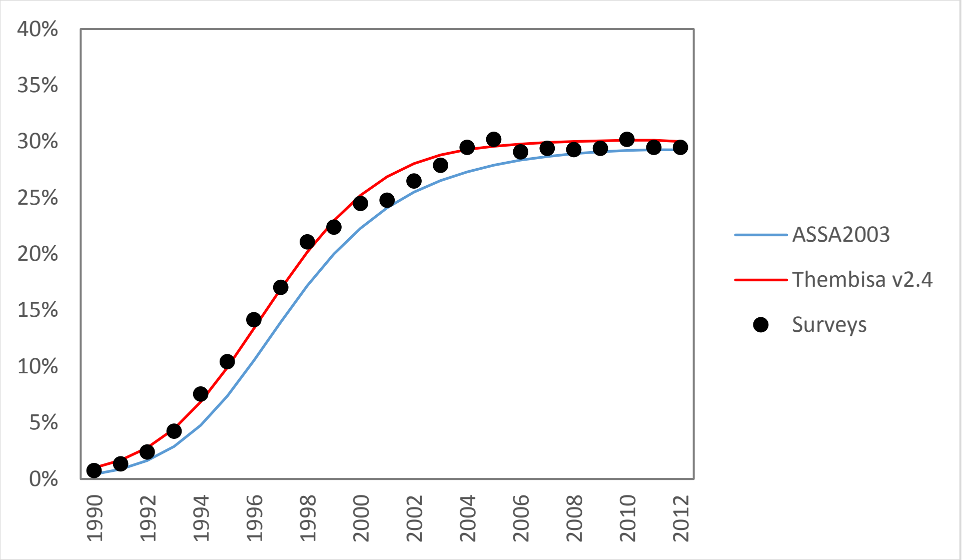

Note: ASSA2003 deliberately under-estimated HIV prevalence in the early ANC surveys. This was because at the time it was believed that the antenatal surveys were biased towards urban areas and therefore likely to over-estimate HIV prevalence. Subsequent work has suggested that the extent of the bias in the early antenatal surveys is in fact very small.

2.2 Population initiating ART

One area of uncertainty in all HIV transmission models was the rate of uptake of ART by people with CD4 cell counts beyond the currently existing CD4 cell count threshold. Uptake can be reduced as a result of demand-side factors, as people with higher CD4 cell counts might not feel as sick as those with lower and are thus less likely to present to facilities for testing and/ or treatment initiation. It could also be a result of supply-side factors such as increasing overcrowding of facilities due to higher numbers of people on treatment, reduced staff time per patient visit, and potentially lower care quality [12,13].

For the analysis of the **2010 WHO guidelines**, the number of individuals starting ART per year in the baseline scenario was assumed to be 80% of those newly eligible for ART from 2009/10 onward in line with the South African National Strategic Plan for HIV and AIDS & STIs for 2007-2011 [14], of whom 90% were assumed to be treated in the public sector. In the Full WHO Guidelines scenario we assumed that the rate of ART initiation in adults with CD4 cell counts of 200-350 cells/microl was 30% of the rate of ART initiation in adults with CD4 cell counts <200 cells/microl. This assumption was based on the CD4 cell count profile in people starting ART in the Aid for AIDS programme, compared to the CD4 cell count distribution in the general population [15]. The Aid for AIDS programme is a South African medical aid programme that started providing ART in the private sector in 2001, well before the public-sector roll-out in 2004. Protocol was to start ART in all patients with CD4 cell counts of <350 cells/microl. During 2001-2002, only 67% of patients initiated ART with CD4 cell counts of <200 cells/microl, with the rest initiating with CD4 cell counts between 200 and 350 cells/microl. In the same time period, the proportion of the untreated HIV-positive population in the <200 cells/microl and 200-350 cells/microl categories were 13% and 24% respectively.

If r is the rate of ART initiation in the CD4 <200 cells/microl category and x is the relative rate of ART initiation in the CD4 200-350 cells/microl category then

$$0.67 = 0.13r /(0.13r + 0.24*rx)$$

from which it follows that

$$x = (0.13/0.67 - 0.13)/0.24$$

which solves to 27% which we rounded up to 30% for this analysis.

For the analysis of the **2013 WHO guidelines**, as the rate of initiation of people with CD4 cell counts between 350 and 500 cells/µl was unknown, for each scenario we modelled three different paces of scale-up that differed in rates of ART initiation and time between diagnosis and ART initiation: Basic scale-up, slow and rapid scale-up (Table 2). The results presented on the paper are those for ‘basic scale-up’.

S1 Table: Scenarios of analysis for 2013 guidelines

**Scenario**	**Relative rate of ART initiation once eligibility criteria are met**				**Average time between diagnosis and ART initiation**[1]
**CD4 cell count stratum [cells/µl]**	**>500**	**350-499**	**200-349**	**<200**	
Basic scale-up	0.4	0.5	0.7	1	6 months
Rapid scale-up	0.7	0.8	0.9	1	3 months
Slow scale-up	0.2	0.4	0.5	1	12 months

[1] This is the average rate for people with CD4 cell counts who hadn’t started ART immediately after diagnosis.

2.3 Early Paediatric Treatment

The number of HIV-positive children requiring Early Paediatric Treatment in the analysis of the 2010 guidelines (New Guidelines and the Full WHO Guidelines scenarios) was calculated based on the assumed uptake of HIV testing in pregnancy, PMTCT coverage and effectiveness, and the proportion of HIV-positive mothers assumed to be breastfeeding. For this, all PMTCT was assumed to be dual therapy comprised of single dose NVP intrapartum to mother and single dose NVP immediately postnatally to child plus AZT for twelve weeks antenatally and single dose intrapartum to the mother, plus AZT one week postnatally to both mother and child. Coverage of children with Early Paediatric Treatment was assumed to increase from 55% in 2010/11 to 85% in 2016/17.

2.4 Rates of death, loss to follow-up, and transition between CD4 cell count strata

For the smoothing of estimated probabilities of death, loss and treatment failure within a given CD4 and time-on-treatment stratum, we first fit a simple linear regression model with the estimated probabilities (stratified by CD4 count) as the dependent variable and time (fit as a quadratic line by also including time squared) as the independent variable. This gave us a predicted probability for each time point and CD4 count stratum. The actual estimated probability from the data was then shrunk towards the predicted value as a function of the variance of the estimate using an Empirical-Bayes shrinkage estimator [16]. This resulted in estimated probabilities with low variance being far from the predicted curve and those with high variance being very close to the fitted line. Figure 2 summarises the impact of this fitting procedure on rates of mortality, loss to follow-up and treatment failure in selected adult health states.

S2 Figure: Selected results of linear regression and Empirical-Bayes shrinkage for time-dependent rates *of (A) mortality, (B) loss to follow up (LTF) and (C) first-line treatment failure, all for adults with CD4 cell counts 350-500 cells/microl. Error bars around observed estimates represent 95% confidence intervals.*

A

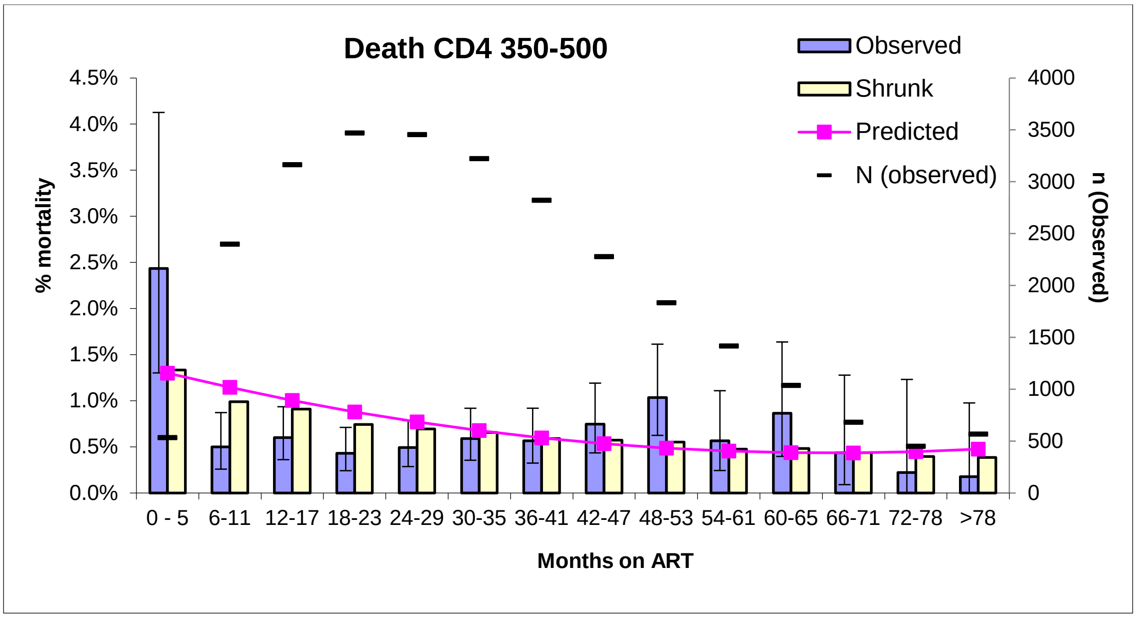
Death CD4 350-500
Observed
Shrunk
Predicted
N (observed)
% mortality
4.5%
4.0%
3.5%
3.0%
2.5%
2.0%
1.5%
1.0%
0.5%
0.0%
n (Observed)
4000
3500
3000
2500
2000
1500
1000
500
0
0 - 5
6-11
12-17
18-23
24-29
30-35
36-41
42-47
48-53
54-61
60-65
66-71
72-78
>78
Months on ART

B

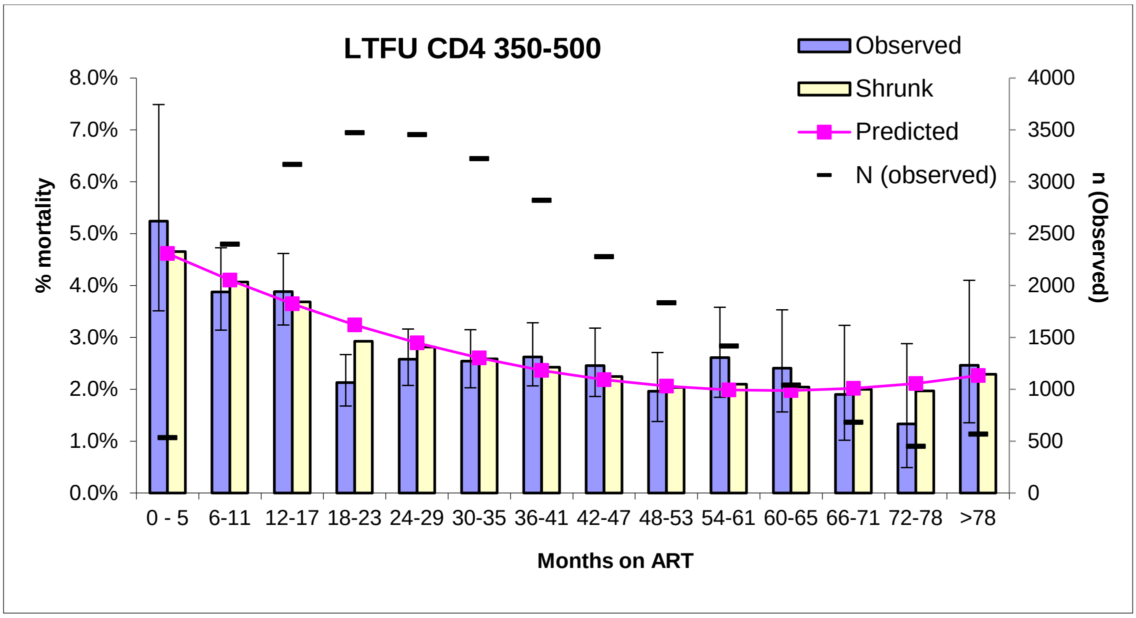

C

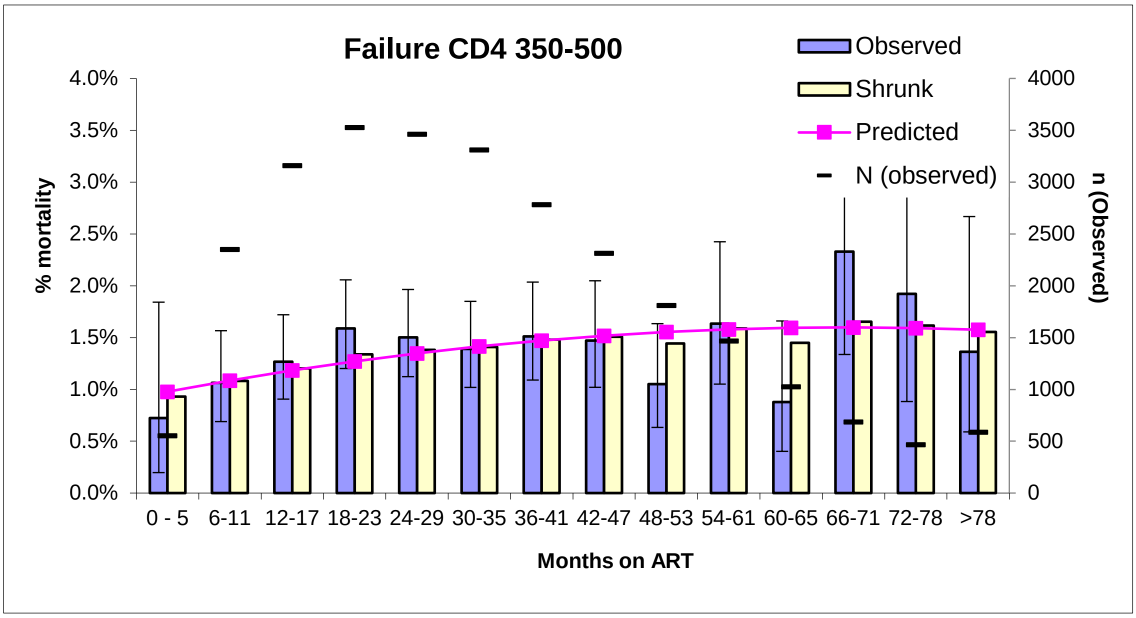

The resulting rates of mortality and LTFU and transitions between health states can be found in Table 3 (adults) and Table 4 (children).

S2 Table: Probabilities of death, loss to follow-up, and treatment failure and transition probabilities between CD4 cell-count defined health states *per 6-month cycle, by type of treatment, CD4 cell count stratum, and (for first-line ART) time on treatment (Adults)*

First-line ART (Adults)							
**Months on first-line ART**	**6-month probability of**			**Probability of transition to CD4 cell count stratum:**			
	**Death**	**Loss to follow-up**	**Treat-ment failure**	**<50 cells/mm^3**	**50-199 cells/mm^3**	**200-350 cells/mm^3**	**>350 cells/mm^3**
**if CD4 cell count >350 cells/mm^3**							
**1 - 6**	1.6%	5.4%	0.5%	0%	2.9%	10.7%	86.4%
**7-12**	1.6%	4.2%	0.8%	0%	1.9%	17.7%	80.4%
**13-18**	1.6%	3.2%	0.9%	0.2%	0.9%	10.4%	88.5%
**19-24**	1.6%	2.0%	0.9%	0.07%	0.5%	9.6%	89.8%
**25-30**	0%	1.6%	1.0%	0.07%	1.3%	9.5%	89.2%
**31-36**	0%	1.2%	0.8%	0%	0.7%	8.0%	91.3%
**37-42**	0.1%	0.6%	0.6%	0.2%	0.5%	9.2%	90.1%
**43-48**	0.1%	0.6%	0.5%	0.3%	0.5%	7.5%	91.8%
**>48**	0%	0.4%	0.3%	0.1%	0.9%	5.4%	93.6%
**if CD4 cell count 200-350 cells/mm3**							
**1 - 6**	1.4%	5.1%	0.1%	0.7%	8.4%	57.0%	33.8%
**7-12**	1.0%	3.6%	0.9%	0.3%	7.8%	62.3%	29.7%
**13-18**	0.4%	2.9%	1.4%	0.2%	5.4%	57.2%	37.3%
**19-24**	0.5%	2.3%	1.6%	0.07%	5.2%	63.2%	31.5%
**25-30**	0.3%	1.9%	1.5%	0.09%	5.8%	63.8%	30.3%
**31-36**	0.3%	1.6%	1.6%	0%	4.5%	63.9%	31.6%
**37-42**	0%	1.4%	1.6%	0%	5.1%	61.3%	33.6%
**43-48**	0.2%	1.3%	1.0%	0%	5.9%	56.3%	37.9%
**>48**	0.2%	1.1%	0.7%	0%	5.4%	56.9%	37.6%
**if CD4 cell count 50-199 cells/mm^3**							
**1 - 6**	2.6%	6.5%	0.3%	1.0%	39.9%	45.3%	13.8%
**7-12**	1.7%	4.7%	1.3%	0.9%	56.0%	39.0%	4.2%
**13-18**	1.2%	4.0%	1.7%	1.7%	52.7%	41.8%	3.8%
**19-24**	1.1%	3.3%	2.1%	0.9%	52.9%	42.9%	3.4%
**25-30**	0.7%	3.1%	2.8%	1.2%	55.2%	41.3%	2.3%
**31-36**	0.5%	2.5%	3.2%	0.5%	54.8%	38.6%	6.1%
**37-42**	0.4%	2.8%	3.4%	0%	54.3%	38.8%	6.9%
**43-48**	0%	3.0%	4.2%	0%	50.9%	43.6%	5.5%
**>48**	0%	3.2%	4.9%	0%	54.3%	31.4%	14.3%
**if CD4 cell count <50 cells/mm^3**							
**1 - 6**	8.0%	9.7%	0.6%	11.6%	71.2%	15.0%	2.2%
**7-12**	5.9%	7.5%	1.2%	23.6%	65.2%	9.4%	1.7%
**13-18**	5.8%	6.0%	2.4%	31.4%	45.1%	19.6%	3.9%
**19-24**	5.3%	4.8%	0.0%	29.4%	50.0%	11.8%	8.8%
**25-30**	0%	4.1%	7.8%	25.0%	58.3%	8.3%	8.3%
**31-36**	0%	0%	0.0%	25.0%	50.0%	25.0%	0%
**37-42**	3.8%	6.6%	0.0%	0%	33.3%	33.3%	33.3%
**43-48**	0%	9.0%	0.0%	0%	33.3%	33.3%	33.3%
**>48**	0%	0%	0.0%	0%	33.3%	33.3%	33.3%

First-line treatment failure (Adults)						
Probability of			Probability of transition to CD4 cell count stratum:			
Death	Loss to follow-up	Switching to second line	<50 cells/mm^3	50-199 cells/mm^3	200-350 cells/mm^3	>350 cells/mm^3
if CD4 cell count >350 cells/mm^3						
0%	2.9%	81.7%	0%	2.6%	17.4%	80.0%
if CD4 cell count 200-350 cells/mm^3						
0.8%	5.5%	77.3%	0%	16.2%	61.9%	21.9%
if CD4 cell count 50-199 cells/mm^3						
1.3%	5.0%	76.8%	7.7%	66.3%	24.0%	1.9%
if CD4 cell count <50 cells/mm^3						
2.8%	7.0%	75.7%	32.3%	58.1%	9.7%	0%

Second-line ART (Adults)					
Probability of		Probability of transition to CD4 cell count stratum:			
Death	Loss to follow-up	<50 cells/mm^3	50-199 cells/mm^3	200-350 cells/mm^3	>350 cells/mm^3
if CD4 cell count >350 cells/mm^3					
0.4%	0.3%	0.6%	0.0%	13.6%	85.8%
if CD4 cell count 200-350 cells/mm^3					
0.7%	0.5%	1.0%	10.6%	59.6%	28.8%
if CD4 cell count 50-199 cells/mm^3					
1.2%	0.5%	4.0%	61.8%	30.2%	4.0%
if CD4 cell count <50 cells/mm^3					
5.1%	1.3%	34.6%	50.0%	11.5%	3.8%

S3 Table*: *Probabilities of death, loss to follow-up, and treatment failure and transition probabilities between CD4 percentage-defined health states per 6-month cycle, by type of treatment and CD4 percentage stratum (Children)

First-line ART (Children < 12 months)						
Probability of			Probability of transition to CD4 % stratum:			
Death	Loss to follow-up	Treatment failure	<5	5-20	21-35	>35
if CD4 % >35						
7.1%	0%	0%	0%	0%	55.6%	44.4%
if CD4 % 21-35						
2.3%	0.8%	0%	0.6%	14.3%	80.0%	5.7%
if CD4 % 5-20						
5.2%	0%	0%	0%	43.8%	49.3%	6.9%
if CD4 % <5						
15.6%	0%	0%	0%	70.0%	30.0%	0%

First-line ART (Children 1 – 5 years)						
Probability of			Probability of transition to CD4 % stratum:			
Death	Loss to follow-up	Treatment failure	<5	5-20	21-35	>35
if CD4 % >35						
2.0%	0.2%	0.9%	0.3%	0.6%	32.2%	66.9%
if CD4 % 21-35						
0.5%	0.2%	1.0%	0.1%	5.6%	78.8%	15.4%
if CD4 % 5-20						
0.2%	0.3%	0.5%	0.7%	48.3%	48.1%	2.9%
if CD4 % <5						
0%	1.3%	0.5%	15.2%	78.1%	6.7%	0%

First-line treatment failure (Children 1 – 5 years)							
**Probability of**			**Probability of transition to CD4 % stratum:**				
**Death**	**Loss to follow-up**	**Switching to second line**	**<5**	**5-20**	**21-35**	**>35**	
**if CD4 % >35**							
0.005%	0.005%	80%	0%	0%	40.0%	60.0%	
**if CD4 % 16-35**							
0.005%	0.005%	80%	1.6%	10.9%	79.7%	7.8%	
**if CD4 % 5-15**							
0.005%	0.005%	80%	0%	70.8%	29.2%	0.0%	
**if CD4 % <5**							
0.005%	0.005%	80%	100.0%	0%	0%	0%	

First-line ART (Children 6 – 13 years)						
**Probability of**			**Probability of transition to CD4 % stratum:**			
**Death**	**Loss to follow-up**	**Treatment failure**	**<5**	**5-20**	**21-35**	**>35**
**if CD4 % >35**						
0.2%	0.6%	0.8%	0%	0%	26.4%	73.6%
**if CD4 % 16-35**						
0.1%	0%	0.5%	0%	1.7%	90.5%	7.8%
**if CD4 % 5-15**						
0.1%	0.5%	0.9%	1.0%	33.8%	64.7%	0.6%
**if CD4 % <5**						
1.5%	0.8%	1.0%	22.5%	62.8%	14.2%	0.5%

First-line treatment failure (Children 6 – 13 years)						
**Probability of**			**Probability of transition to CD4 % stratum:**			
**Death**	**Loss to follow-up**	**Switching to second line**	**<5**	**5-20**	**21-35**	**>35**
**if CD4 % >35**						
0%	0.005%	80%	0%	0%	26.7%	73.3%
**if CD4 % 16-35**						
0%	0.005%	80%	0%	3.6%	92.3%	4.1%
**if CD4 % 5-15**						
0%	0.005%	80%	9.0%	61.2%	29.9%	0.0%
**if CD4 % <5**						
0%	0.005%	80%	16.7%	83.3%	0%	0%

Second-line ART (Children 6 – 13 years)					
**Probability of**		**Probability of transition to CD4 % stratum:**			
**Death**	**Loss to follow-up**	**<5**	**5-20**	**21-35**	**>35**
**if CD4 % >35**					
0%	0.7%	0%	0%	100.0%	0%
**if CD4 % 16-35**					
0%	0.7%	0%	6.4%	87.2%	6.4%
**if CD4 % 5-15**					
0%	0.7%	0%	66.7%	33.3%	0%
**if CD4 % <5**					
0%	0.7%	100.0%	0%	0%	0%

2.5 Changes between drug regimens

We also calculated rates of toxicity development requiring single-drug substitution of d4T with TDF and of TDF with AZT used in the New Guidelines scenario (Table 5). Estimates of mortality, LTFU, and treatment failure rates amongst paediatric patients (≤13) were based on data from a cohort of 3,748 paediatric patients accessing ART at Harriet Shezi Children's Clinic (HSSC), Chris Hani Baragwanath Hospital, Johannesburg, between April 2004 and May 2009. Children ≤3 receive d4T,

3TC and LPV/r as first line and AZT, ddI and either NVP or EFV as second line; children >3 receive the same regimens as adults.

S4 Table: Assumptions regarding drug toxicity

Parameter	Value		Source
Incidence of severe d4T toxicity in adults (per number of months on treatment) (New Guidelines + Full WHO Guidelines)	0 - 5	0.0399	TLC data, based on [17]
	6-11	0.1067	
	12-17	0.1333	
	18-23	0.0621	
	24-29	0.0289	
	30-35	0.0135	
	36-41	0.0063	
	42-47	0.0029	
	> 47	0.0014	
Incidence of severe renal failure under TDF (per number of months on treatment) (New Guidelines + Full WHO Guidelines)	0 - 6	0.0171	TLC data
	> 6	0.0158	

REFERENCES

1. World Health Organization: Rapid advice: antiretroviral therapy for HIV infection in adults and adolescents. Geneva 2009
2. World Health Organization: Antiretroviral Therapy for HIV Infection in Adults and Adolescents: Towards Universal Access. Recommendations for a public health approach. 2010 revision. Geneva, July 2010
3. National Department of Health, South Africa: National Antiretroviral Treatment Guidelines. First edition. Pretoria, April 2004.
4. Clinton Foundation Antiretroviral (ARV) Price List, version August 2009
5. World Health Organization. Consolidated guidelines on the use of antiretroviral drugs for treating and preventing HIV infection. Recommendations for a public health approach. Geneva, June 2013
6. Adam MA, Johnson LF: Estimation of adult antiretroviral treatment coverage in South Africa. S Afr Med J 2009; 99: 661-7
7. Johnson L: THEMBISA version 1.0: A model for evaluating the impact of HIV/AIDS in South Africa. Centre for Infectious Disease Epidemiology and Research working paper. February 2014
8. Johnson LF, Chiu C, Myer L, Davies M-A, Dorrington RE, Bekker L-G, Boulle A, Meyer-Rath G: Prospects for HIV control in South Africa: a model-based analysis. Global Health Action 9:30314 (2016)
9. Department of Health, South Africa, and South African National AIDS Council: South African HIV and TB Investment Case - Reference Report Phase 1. March 2016.
10. Department of Health, South Africa, and South African National AIDS Council: South African HIV and TB Investment Case - Summary Report Phase 1. March 2016.
11. Chiu C, Johnson LF, Jamieson L, Larson BA, Meyer-Rath G: Designing an optimal HIV programme for South Africa: Does the optimal package change when diminishing returns are considered? BMC Public Health 17:143. DOI 10.1186/s12889-017-4023-3 (2017)
12. Meyer-Rath G, Over M: Modelling the Cost of Antiretroviral Treatment: State of the Art and Future Directions. PLoS Med 9(7):e1001247. doi: 10.1371/journal.pmed.1001247 (2012)
13. Mikkelsen E, Hontelez JAC, Jansen MPM, Baernighausen T, Hauck K, Johansson KA, Meyer-Rath G, Over M, de Vlas SJ, van der Wilt G, Tromp N, Bijlmakers L, Baltussen RMPM: Evidence

for scaling up HIV treatment in sub-Saharan Africa: A call for incorporating health system constraints. PLoS Med 14(2): e1002240.doi:10.1371/journal.pmed.1002240 (2017)

14. Cleary S: The costs of the National Strategic Plan on HIV and AIDS & STIs 2007-2011. Pretoria 2007
15. Nachega JB, Hislop M, Dowdy DW, et al: Adherence to Highly Active Antiretroviral Therapy Assessed by Pharmacy Claims Predicts Survival in HIV-Infected South African Adults. J Acquir Immune Defic Syndr 2006;43:78-84
16. Greenland S, Robins JM: Empirical-Bayes adjustments for multiple comparisons are sometimes useful. Epidemiology 1991;2:244-51
17. Rosen S, Long L, Fox M, Sanne I: Cost and cost-effectiveness of switching from stavudine to tenofovir in first-line antiretroviral regimens in South Africa. J Acquir Immune Defic Syndr 2008;48(3):334-44.
